# Isolation and evaluation of the pathogenicity of a hybrid shiga toxin-producing and Enterotoxigenic *Escherichia coli* in pigs

**DOI:** 10.1186/s12917-024-04317-z

**Published:** 2024-10-21

**Authors:** Danaya Nammuang, Yi-Wen Shen, Chiao-Hsu Ke, Nan-Ling Kuan, Chao-Nan Lin, Kuang-Sheng Yeh, Yen-Chen Chang, Chia-Yu Chang, Hui-Wen Chang

**Affiliations:** 1 Graduate Institute of Molecular and Comparative Pathobiology, School of Veterinary Medicine, National Taiwan University, No. 1, Section 4, Roosevelt Rd, Taipei, 10617 Taiwan; 2 Sustainable Swine Research Center, National Pingtung University of Science and Technology, Pingtung, Taiwan; 3 Animal Disease Diagnostic Center, College of Veterinary Medicine, National Pingtung University of Science and Technology, Pingtung, 91201 Taiwan; 4 Department of Veterinary Medicine, School of Veterinary Medicine, National Taiwan University, Taipei, 10617 Taiwan; 5 Biology Division, Veterinary Research Institute, Ministry of Agriculture, Tamsui, New Taipei City, Taiwan; 6 Department of Veterinary Medicine, College of Veterinary Medicine, National Pingtung University of Science and Technology, Pingtung, 91201 Taiwan; 7 College of Veterinary Medicine, Department of Veterinary Medicine, National Chung Hsing University, Taichung, 402 Taiwan

**Keywords:** Hybrid STEC/ETEC, Enterotoxigenic *E. Coli*, Post-weaning diarrhea

## Abstract

**Background:**

Porcine pathogenic *Escherichia coli* (*E. coli*), the globally recognized important pathogen, causes significant economic loss in the field. Enterotoxigenic *E. coli* (ETEC) causes porcine neonatal and post-weaning diarrhea (PWD), frequently carrying F4 adhesin, F18 adhesin, Heat-Stable toxin (ST), and Heat-Labile toxin (LT). Shiga Toxin-Producing *E. coli* (STEC) produces F18 adhesin and Shiga toxin type 2e (*stx2e*), majorly leading to systemic endothelial cell damage and edema disease. In this study, hemolytic pathogenic hybrid STEC/ETEC strains carrying ST and LT genes of ETEC and the *Stx2e* gene of STEC isolated from pigs with PWD in Taiwan were identified. The pathogenicity of a Taiwan hybrid STEC/ETEC strain was evaluated by oral inoculation in post-weaning pigs.

**Results:**

Next generation sequencing and multilocus sequence typing of two hybrid Taiwan porcine STEC/ETEC isolates indicated that these two isolates were closely related to the ST88 porcine hybrid STEC/ETEC isolated from pigs with watery diarrhea. Furthermore, the two hybrid Taiwan porcine STEC/ETEC isolates also displayed combinations of multiple resistance genes encoding mechanisms for target modification and antibiotic inactivation. Animal experiments confirmed that the Taiwan hybrid STEC/ETEC could cause watery diarrhea in post-weaning pigs with no signs of edema disease and minimal histopathological lesions.

**Conclusion:**

To the best of the authors’ knowledge, the present study is the first study demonstrating intestinal pathogenicity of the hybrid STEC/ETEC in pigs. The result suggests that the hybrid STEC/ETEC should be considered as a new emerging pathogen and a new target for vaccine development.

**Supplementary Information:**

The online version contains supplementary material available at 10.1186/s12917-024-04317-z.

## Background

*Escherichia coli* (*E. coli*) infections in the swine industry are recognized as an important disease worldwide. Infections usually occur during the neonatal and post-weaning periods, cause increased mortality and morbidity, and lead to retarded growth [1]. It affects the economic status of swine farms by increasing the cost of veterinarians, workers, antimicrobial drugs, and other treatments. Moreover, the use of antimicrobial drugs in farms can cause multidrug resistance and antimicrobial residues in the environment. Recently, newly emerging pathogenic *E. coli* from swine farms in Japan showed higher multidrug resistance, especially resistant to fluoroquinolones [2]. Furthermore, multidrug-resistant bacteria can be transferred from farms to slaughterhouse and pose a risk of contaminating meat [3].

Porcine pathogenic *E. coli* strains can be categorized based on their virulence factors. Enterotoxigenic *E. coli* (ETEC) causes watery diarrhea in neonatal and post-weaning pigs. When piglets ingest sufficient ETEC, bacteria attach to enterocytes at the mid-jejunum to the ileum by adhesins, such as F4 or F18. After multiplication, these bacteria produce exotoxins such as Heat-Labile toxin (LT) and/or Heat-Stable toxin (ST). LT upregulates cyclic adenosine monophosphate (cAMP), and ST induces cyclic guanosine monophosphate (cGMP) in intestinal epithelial cells, leading to the hypersecretion of water in the intestinal lumen [4] and yellow or gray watery stool, dehydration, and emaciation in affected pigs. However, STEC causes edema disease in post-weaning pigs by producing Shiga toxin type 2e (Stx2e). The toxin is absorbed into circulation, binds to globotetraosylceramide in endothelial cells, and inactivates ribosomes, causing apoptosis, capillary damage, and fluid loss [5]. Piglets with edema disease commonly die suddenly without signs of sickness and develop eyelids and forehead swelling. Occasionally, neurological signs can be observed. Enteropathogenic *E. coli* (EPEC) also causes watery diarrhea in neonatal and post-weaning piglets. In contrast to ETEC and STEC, EPEC use a bacterial outer membrane protein, known as AE factor eae or intimin, to attach to the intestinal epithelial cells. However, other diarrheal mechanisms of EPEC remain unclear.

Several pathogenic *E. coli* isolates concurrently carry virulence factors of ETEC and STEC in humans, livestock (goats, sheep, and cattle), poultry (chicken and ducks), wild ruminants (moose, reindeer), swine, wild boar, and humans [6–11]. There are several terms for these strains, such as “hybrid,” “blended virulence profiles,” “virulence combination,” or “hetero” [7, 12–14]. These hybrid strains have been isolated from humans with Hemolytic Uremic Syndrome (HUS) or diarrhea [7]. Hybrid strains have been isolated from pigs with post-weaning diarrhea and pigs with edema disease in South Korea, China, and Italy [8, 15, 16]. In addition, hybrid STEC/ETEC has been isolated from wild boars with edema disease in France [11].

To characterize pathogenic *E. coli* in Taiwan, multiplex polymerase chain reaction (PCR) and PCR were used to determine the presence of virulent genes in the genomes of ten pathogenic *E. coli* collected from diseased pigs between 2017 and 2023. A hybrid STEC/ETEC isolate was orally inoculated in post-weaning pigs, and the pathogenicity was evaluated by daily observation of fecal consistency, fecal bacterial shedding, and pathology evaluation.

## Results

### PCR-based virulence factors profiling of *E. Coli* strains

Ten pathogenic *E. coli* isolates exhibiting beta-hemolysis were obtained. While hemolytic *E. coli* strains that showed *sta*, *stb*, and *lt* genes were considered ETEC [17–19], *stx2e* were considered STEC [19], with *st* and/or *lt* and the *stx2e* genes were considered as hybrid STEC/ETEC strains [7], and *eae* gene (intimin) were identified as EPEC, five of ten (50%) *E. coli* isolates were ETEC, one was STEC, one was EPEC, and, interestingly, three of ten (30%) were hybrid Taiwan porcine STEC/ETEC strains carrying *st* and *lt* genes of ETEC and the *stx2e* gene of STEC based on the multiplex PCR results. The results were summarized in Table 1.

**Table 1:** History and virulence PCR profiling of hemolytic *E. Coli* isolated from 2017 to 2023

No. (farm)	Type of strains	Age of pigs	Origin	Sign	Isolation year	Virulence gene							
						K88 (F4)	F18	F41	LT	STa	STb	STx2e	Intimin (Eae)
**1** (2675)	Hybrid STEC/ ETEC	5–6 weeks	Small intestine	Watery diarrhea	2017	-	+	-	+	+	+	+	-
**2** (191 H)	ETEC	5–6 weeks	Brain	Dyspnea	2020	-	+	-	+	-	+	-	-
**3** (272 H)	Hybrid STEC/ ETEC	3–4 weeks	Intestine	Watery diarrhea	2020	-	+	-	+	+	+	+	-
**4** (295 H)	STEC	3–4 weeks	Fecal swab	Bloody diarrhea	2020	-	-	-	-	-	-	+	-
**5** (088)	Hybrid STEC/ ETEC	4 weeks	Intestine	Watery diarrhea	2021	-	+	-	+	+	+	+	-
**6** (112)	ETEC	4 weeks	Small intestine	Watery diarrhea	2022	-	-	-	-	-	+	-	-
**7** (149)	ETEC	1 week	Small intestine	Watery diarrhea	2022	+	-	-	-	+	+	-	-
**8** (161)	EPEC	4 weeks	Small intestine	Watery diarrhea	2022	-	-	+	-	+	-	-	+
**9** (344)	ETEC	4 weeks	Small intestine	Watery diarrhea	2022	-	-	-	+	-	+	-	-
**10** (387)	ETEC	9 weeks	Small intestine	Watery diarrhea	2023	-	-	-	+	-	+	-	-

### Next-generation sequencing of a hybrid Taiwan Porcine STEC/ETEC isolate

To confirm the identification of the hybrid Taiwan porcine STEC/ETEC isolates, next-generation sequencing (NGS) was subsequently performed in two hybrid Taiwan porcine STEC/ETEC isolates, 2675 (No.1 in the Table 1) and 088 (No.5 in Table 1) exhibiting identical virulence PCR-positive profiles for *stx2*e, *F18*, *lt*, and *sta*, and *stb*. The NGS results of the hybrid STEC/ETEC isolate 2675 (No.1) and 088 (No.5) were deposited to the NCBI Sequence Read Archive (SRA) under the accession number: PRJNA1123447 (SAMN41811493) and (SAMN41811492), respectively.

### Multilocus sequence typing (MLST)

The sequence type of two hybrid Taiwan porcine STEC/ETECs was determined based on the *E. coli* multilocus sequence typing scheme. The hybrid STEC/ETEC isolate 2675 (No.1) and 088 (No.5) were both classified as ST88 with 100% identity in the *adk*, *fumC*, *gyrB*, *icd*, *mdh*, *purA*, and *recA* genes.

### Antimicrobial susceptibility and resistome analysis of the hybrid Taiwan Porcine STEC/ETEC isolate

Using antimicrobial susceptibility tests, the hybrid STEC/ETEC isolate 2675 (No.1) exhibited resistance to 29 out of the 34 antimicrobial agents tested (supplementary Table 3), excluding amikacin, imipenem, meropenem, nitrofurantoin, and polymyxin B. In the resistome analysis, the hybrid Taiwan porcine STEC/ETEC isolate 2675 (No.1) displayed combinations of resistance genes encoding mechanisms for target modification and antibiotic inactivation (Table 2). The hybrid STEC/ETEC isolate 88 (No. 5) also exhibited resistance to most of antimicrobial agents tested (supplementary Table 4), excluding imipenem, the third-generation cephalosporin antibiotic, cefoperazone, and the fourth-generation cephalosporin antibiotic, cefquinome. These two hybrid ETEC/STEC isolates harboured multiple β-Lactamase resistance genes, including the *bla*_*TEM−B1*_, *bla*_*oxa−1*_, and *bla*_*CMY−2*_ genes; various folate pathway antagonist resistance genes, consisting the *sul3* and *dfrA12* genes; and aminoglycoside resistance genes such as the *aac* [3] or *aac(6’)* genes; and several other resistance genes donated to be against variable antibiotics (Table 2).

**Table 2:** Overview of resistome genes in hybrid STEC/ETEC E. Coli isolates

Isolate	MLST	β-Lactamase Resistance Genes			Aminoglycoside Resistance Genes	Folate pathway antagonist Resistance Genes	Other Resistance Genes
		TEM	OXA	CMY			
No.1 (2675)	ST88	*TEM-B1*	*-*	*CMY-2*	*aac(3)*	*sul3 dfrA12*	*floR cmlA1 mef(B) mcr-1.1 tet(A)*
No.5 (088)	ST88	*-*	*OXA-1*	*CMY-2*	*aac(3) aac(6’)*	*sul1 sul2 sul3 dfrA12*	*cmlA1 catB3 floR mph(A) tet(M) tet(B)*

### Pathogenicity of the hybrid STEC/ETEC isolate 2675 (No.1) in pigs

**Fig. 1:**
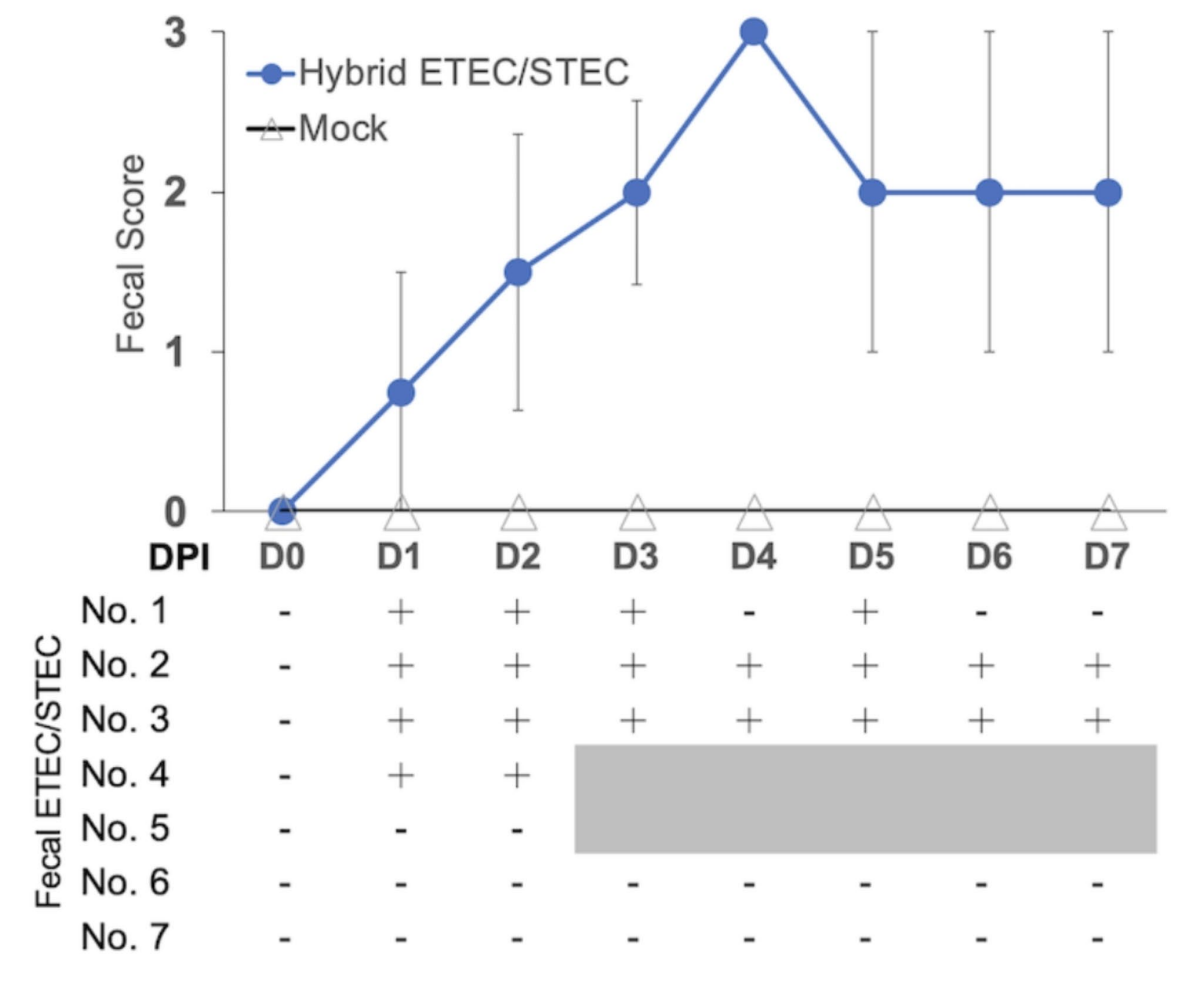
Daily fecal scores and fecal hybrid ETEC/STEC detection by using multiplex virulence profiling PCR in the Taiwan porcine hybrid ETEC/STEC 2675 (No.1) -inoculated pigs. Before inoculation mock- and hybrid ETEC/STEC 2675 (No.1)-inoculated pigs were orally received 60 ml of 1.4% sodium bicarbonate in distilled water. Scores of 0, 1, 2, and 3 indicate normal, soft feces, semi-liquid, and liquid, respectively. The pig 4 exhibiting severe diarrhea together with the pig No. 5 in the mock-inoculated group were euthanized at 2 days post inoculation (DPI) for pathological examination. + virulence genes of hybrid ETEC/STEC positive; - virulence genes of hybrid ETEC/STEC negative

In the present study, the pathogenicity of the hybrid STEC/ETEC isolate 2675 (No.1) was evaluated in 4-week-old weaning pigs. While all pigs in the mock-inoculated group presented normal fecal consistency scores and were healthy during the experimental period (Fig. 1), all pigs in the hybrid STEC/ETEC-inoculated group showed severe watery diarrhea (score 3) at different time points, including one at 1 DPI, two at 2 DPI, and three at 4 DPI. Most pigs in the hybrid STEC/ETEC-inoculated group constantly exhibited a score of 3 after showing a watery diarrheal sign, except pig No. 3, which revealed a score of 3 at 4 DPI and recovered with normal feces (score 0) at 5 DPI. Because of No. 4, which first appeared with watery diarrhea (score of 3) exhibiting severe weakness and dehydration, the animal was euthanized at 2 DPI according to the IACUC guidelines, and a full necropsy was performed to evaluate the pathology. At the same time, a pig from the mock-inoculated group was randomly selected and euthanized for pathological comparison. Pigs in both groups were sacrificed at 7 DPI for pathological evaluation and bacterial re-isolation. At necropsy, dilation of the small intestine with watery intestinal content was noted in the hybrid STEC/ETEC-inoculated pigs, whereas intestinal contents were normal, and no significant lesions were observed in mock-inoculated pigs. Histopathological findings of all pigs showed no lesion and no gram-negative rod bacteria on villous epithelial cells in either group (data not shown).

### Detection of hybrid STEC/ETEC in feces

Fecal shedding of the hybrid STEC/ETEC isolate 2675 (No.1) was evaluated daily for seven days. Using multiplex PCR, detectable hybrid STEC/ETEC isolates were first observed in all hybrid STEC/ETEC-inoculated animals at 1 DPI and persistent during the whole experimental period, except pig No.1, which showed intermittent bacterial shedding after 3 DPI. No detectable hybrid STEC/ETEC were identified in the feces of mock-inoculated pigs (Fig. 1). Bacterial isolation was also performed during the necropsy. The hybrid STEC/ETEC exhibiting beta-hemolysis and virulence genes detected by multiplex PCR was identified in the small intestinal content of all hybrid STEC/ETEC-inoculated pigs. No beta-hemolytic hybrid STEC/ETEC was identified in the mock-inoculated pigs.

## Discussion

Porcine hybrid STEC/ETEC strains have been isolated from pigs with PWD and pigs with edema disease in several countries [8, 15, 16]. However, the information regarding to the pathogenicity of the hybrid STEC/ETEC in pigs is limited. In this study, we identified three hybrid STEC/ETEC *E. coli* from pigs with PWD in Taiwan. The pathogenicity of a hybrid STEC/ETEC was confirmed in post-weaning pigs based on the finding of severe watery diarrhea with minimal histopathological lesion and the detection and re-isolation of the hybrid STEC/ETEC strain in inoculated pigs. To the authors’ knowledge, this is the first identification and animal experiment confirming the pathogenicity of the hybrid STEC/ETEC in Taiwan. The high prevalence and pathogenicity of hybrid STEC/ETEC demonstrated in the present study suggests that hybrid STEC/ETEC should be considered as an emerging pathogen and a new target for vaccine development.

To characterize pathogenic *E. coli*, multiplex PCR and conventional PCR were used to detect virulence factors of hemolytic *E. coli*. Interestingly, 30% of *E. coli* in our study were hybrid STEC/ETEC. This prevalence is lower than a report in South Korea indicating that 46.8% of F18 *E. coli* isolates from diarrhetic or edema diseases pigs were hybrid STEC/ETEC [8] and higher than the research in China indicating that about 10% *E. coli* isolates from weaned piglets with post-weaning diarrhea and/or edema disease are hybrid STEC/ETEC [15]. The identification of pathogenic hybrid STEC/ETEC in Taiwan herein suggests a requirement of further comprehensive epidemiological surveys of the pathogen.

In the present study, only hemolytic *E. coli* were investigated because pathogenic *E. coli* causing neonatal diarrhea, post-weaning diarrhea, or edema disease generally exhibits hemolysis on blood agar and our result demonstrated that the most isolated *E. coli* were hybrid STEC/ETEC and ETEC; the minority were STEC and EPEC. Correspondingly, investigating swine ETEC in Zimbabwe showed that 100% of F4-positive ETEC and 88.24% of F18-positive ETEC produced hemolysin [20]. In the same way, all F18-positive *E. coli* isolated from diarrheal piglets in South Korea, including ETEC, STEC, and hybrid STEC/ETEC, also showed hemolysis on blood agar [21]. However, the actual number of EPEC can be underestimated since EPEC usually is recognized as nonhemolytic [22–24].

In China, hybrid STEC/ETEC isolates attacking post-weaning pigs majorly contained F18 adhesin and Stx2e [15]. The result is similar to our finding of all three hybrid STEC/ETEC strains isolated from pigs with PWD in Taiwan carrying F18, *lt*, *sta*, *stb*, and *stx2e* virulent genes. Normally, intestinal epithelial cells of piglets express F18 receptors after weaning, and F4 receptors are normally expressed in the neonatal period [25]. Consequently, F18 is important for pathogenic *E. coli* to infect post-weaning piglets. For these reasons, post-weaning pigs were used for the animal study. After inoculation, all pigs in the hybrid STEC/ETEC-inoculated group exhibited typical ETEC-like illness, including severe watery diarrhea, rough coat, fecal stain around the anus, and sunken eyes, without showing clinical signs of edema disease. It was shown that hybrid STEC/ETEC strains could be isolated from pigs and wild boar with diarrheal or edema disease [8, 10, 11, 15, 26, 27], although no animal cohort study was performed in these reports. The absence of edema disease observed in our study may be due to our hybrid STEC/ETEC strain being isolated from pigs with PWD without systemic edema disease. The capability of the hybrid STEC/ETEC to produce Shiga toxin type 2e toxin the existence of other virulence factors responsible for edema diseases, such as enterohemolysin (*ehxA*) and α-hemolysin (*hlyA*), need to be further investigated to help to understand the pathogenesis of hybrid STEC/ETEC [28].

Based on the MLST result, the hybrid Taiwan porcine STEC/ETEC isolate 2675 (No.1) belonged to ST88. It has been demonstrated that nearly all (97.9%, 184 out of 188) of the PFGE cluster III/ST88 strains with the well-conserved VF positive profile of *stx2e*, *lt*, *st*, and F18 were isolated from swine with lethal diarrhea [2]. The pathogenicity of these hybrid ETEC/STEC was similar to hybrid Taiwan porcine STEC/ETEC isolates observed in the present study. Furthermore, it has also been demonstrated that a high level of multidrug resistance in ST88 strains, including cephalosporins, second-generation cephalosporin and cephamycin, and to third-generation cephalosporin [2]. The hybrid Taiwan porcine STEC/ETEC isolate 2675 (No.1) also exhibits resistance to one to third-generation cephalosporins and carries multiple β-Lactamase resistance genes, including the *bla*_*TEM−B1*_and *bla*_*CMY−2*_ genes; various folate pathway antagonist resistance genes, consisting the *sul3* and *dfrA12* genes; and aminoglycoside resistance genes. Our results indicate that the hybrid Taiwan porcine STEC/ETEC isolate isolated from diarrheal pigs has multiple virulence factors and antimicrobial resistance.

In the present study, although the ETEC-like clinical sign of watery diarrhea was observed in all hybrid STEC/ETEC-inoculated pigs, minimal bacterial colonies and inflammation on intestinal epithelial cells were observed. It has been demonstrated that general bacterial rod attachment on the enterocytes in ETEC-inoculated pigs was sporadic, with no apparent correlation with direct bacterial plating rate [29], suggesting that lack of villus lesion in the ETEC challenge study may be due to short trial duration and histological analysis does not appear to be a useful method to evaluate ETEC animal models [29]. The severe watery diarrhea but minimal lesions could be due to the high dosage of the hybrid STEC/ETEC strain, which concurrently produces all four LT, STa, and STb virulent proteins in the inoculum, directly resulting in a severe illness without bacterial colonization of the villi in the present study.

## Conclusions

In summary, our study demonstrated that the majority of pathogenetic hemolytic *E. coli* isolates in Taiwan in this study were ETEC and hybrid STEC/ETEC, and a hybrid STEC/ETEC has also been demonstrated mainly causing severe diarrhea in post-weaning pigs without edema disease based on the clinical signs in the field and the results of experimental inoculation in pigs. Because of the increasing prevalence of hybrid STEC/ETEC, it is important to develop multivalent vaccines against both hybrid STEC/ETEC and ETEC to prevent bacterial diarrhea in pigs.

## Materials and methods

### Samples collection and bacterial identification

Pathogenic *E. coli* strains used in this study were obtained from conventional Duroc x Landrace-Large white pig farms in Taiwan, including Taoyuan, Yunlin, and Yilan counties. These pathogenic *E. coli* isolates were collected from diseased pigs aged between 5 d-old to 9-week-old, exhibiting clinical signs of watery diarrhea or dyspnea (Table 1). Bacteria were isolated from fecal swabs, small intestine, large intestine, or brain using Tryptic Soy Agar with 5% Sheep blood (Dr. Plate Biotech, Taipei, Taiwan) and incubated at 37 °C aerobically overnight. Bacterial colonies were selected for single colonies and re-cultured on Tryptic Soy Agar with 5% Sheep blood. Single bacterial colonies were selected for Gram stain. Bacterial identification and antimicrobial sensitivity assays were performed using the VITEK^®^ 2 Compact (bioMérieux Inc., Durham, USA).

### Preparation of *E. Coli* samples and virulence genes detection

A single colony of *E. coli* isolates was suspended in 50 µl of Hypure™ nuclease-free deionized distilled water (Cytiva, Utah, USA). Virulence factors of *E. coli* were determined by multiplex and conventional PCR using the primers and protocol described in supplementary Tables 1 and 2 [20, 30]. The AmaR OnePCR was used as a PCR reaction mixture (GeneDireX, Taipei, Taiwan). For multiplex PCR, the mixture contained 25 µl of AmaR OnePCR, 1 µl of forward and reward primer sets, 2 µl of DNA template, and nuclease-free deionized distilled water was added to 50 µl of the final volume. For conventional PCR, the mixture contained 10 µl of AmaR OnePCR, 2 µl of forward and reward primer sets, 2 µl of DNA template, and added nuclease-free deionized distilled water to a final volume of 20 µl. Reactions were performed in T100™ Thermal Cycler (Bio-Rad Laboratories, California, USA) for 35 cycles. The DNA amplicons were separated using 1.5% agarose gel electrophoresis, and ethidium bromide was used as a stain to visualize the PCR products.

### Nucleotide sequence analysis

To confirm the virulence factor genes of the *E. coli* isolates, the PCR products were cloned into a 2.1-TOPO™ vector (Thermo Fisher Scientific, California, USA) and transformed into TOP10 competent cells. Competent cells were spread over a Lysogeny broth (LB) agar plate containing 100 µg/ml ampicillin and aerobically incubated at 37 °C overnight. Bacterial colonies were selected for single colonies, cultured in LB broth containing 100 µg/ml ampicillin and incubated at 37 °C overnight at 210 rpm. Recombinant plasmids were isolated using the QIAprep Spin Miniprep Kit (Qiagen GmbH, Hilden, Germany), and target genes were identified by restriction digestion reactions using *Eco*RI-HF^®^ (New England Biolabs, Massachusetts, USA) and sent for sequencing at TRI-I biotech (New Taipei, Taiwan).

### Hybrid STEC/ETEC strains inoculation in post-weaning pigs

Seven post-weaning Duroc x Landrace-Large white male piglets at 4 weeks of age were obtained from a conventional pig farm. No genomes of porcine epidemic diarrhea virus, porcine deltacoronavirus, or virulence genes of hybrid STEC/ETEC, ETEC, Enteropathogenic *E. coli* (EPEC), or STEC in fecal swabs of these pigs were detected by real-time RT-PCR, multiplex PCR and conventional PCR. Non-antibiotic creep feed was provided ad libitum throughout the whole experimental period.

The hybrid STEC/ETEC isolate 2675 (No.1) that was PCR-positive for *stx2e*, F18, *lt*, and *sta*, and *stb* was cultured overnight in sterile LB broth without antibiotics at 37 ^o^C overnight, 210 rpm. The concentration of the bacteria was determined by measuring the optical 10-fold dilutions of the bacterial suspension at 600 nm (OD_600_) with a WPA Biowave II spectrophotometer (Biochrom, Cambridge, United Kingdom). Seven five-week-old pigs were introduced to the animal house from a conventional farm and stochastically separated into two groups: a mock-inoculated group (*n* = 3) and a hybrid STEC/ETEC group (*n* = 4). All piglets orally received 60 ml of 1.4% sodium bicarbonate in distilled water. After 15 min, the pigs in the hybrid STEC/ETEC group were inoculated with 1 × 10^10^ hybrid STEC/ETEC in 10 ml LB broths, whereas pigs in the mock-inoculated group orally received 10 ml of sterile LB. After inoculation, daily clinical fecal scoring and fecal swab collection for bacterial isolation and virulence genes detection by PCR were performed for seven days. Scores of 0, 1, 2, and 3 indicate normal, soft feces, semi-liquid, and liquid, respectively. All experimental procedures on the animal were reviewed and approved by the Institutional Animal Care and Use Committee of National Taiwan University (Taipei, Taiwan, NTU-111-EL-00070).

### Evaluation of the pathogenicity

At 2-day post-inoculation (DPI), the pig No. 4 first appeared with severe watery diarrhea (score 3) together with the pig No. 5 appeared normal were euthanized via electrocution with current of 110 V applied for 10 s for pathological evaluation. At 7 DPI, the rest of the animals were euthanized for pathological evaluation. At necropsy, gross lesions were recorded and tissues from all organs were collected routinely for histopathological examination. All tissue samples were fixed in 10% neutral buffered formalin. Formalin-fixed tissues were embedded in paraffin wax to process formalin-fixed, paraffin-embedded (FFPE) blocks and were cut in 3 μm thickness by a microtome. FFPE tissue sections were stained with hematoxylin and eosin (H&E), Brown and Brenn (B&B), and Warthin-Starry Stain, following standardized protocols. Segments of the jejunum and ileum were collected separately to isolate the bacteria from the small intestine. The isolated bacteria were streaked on Tryptic Soy Agar with 5% Sheep blood and incubated at 37 °C aerobically overnight. Multiplex PCR analyses were used to detect the virulence genes of the challenge strain from hemolytic colonies on blood agar, as per the protocol described above. Gross examination was observed during necropsy and the small intestine was collected for histopathological examination.

### Extraction of genomic DNA and preparation of DNA sequencing libraries

Total genomic DNA was isolated from each sample using a DNA Extraction Kit (Qiagen, Hilden, Germany) according to the manufacturer’s instructions. A Qubit fluorometer was used to measure the concentrations of genomic DNA following the standard protocol (Invitrogen, Carlsbad, CA, USA). For library preparation, all reagents used were provided in the Illumina commercial kits. The Illumina DNA Preparation Kit (Illumina, San Diego, CA, USA) and IDT for Illumina DNA/RNA/ UD indexes Set A (Illumina) were employed for sequencing library preparation following by the manufacturer’s instructions. The quality of the indexed libraries was determined in ng/µL units on a Qubit fluorometer (Invitrogen). Quantitation and visualization were performed using capillary electrophoresis (Bioptic, New Taipei City, Taiwan). The average base pair for each peak location was determined for the samples. Using the formula recommended by Illumina, concentrations were converted to nM values. Each DNA library was normalized and diluted to a final loading concentration of 100 pM. The pooled DNA libraries were loaded onto the iSeq 100 flow cell (Illumina) for sequencing in a total volume of 20 µL. The cartridge (with the preinserted flow cell) was then inserted into the iSeq 100 sequencer (Illumina). Whole-genome sequencing was performed on the sequencer with an approximately 17-hour run time using paired-end sequencing of 2 × 150 bp reads.

### iSeq 100 QC and NGS bioinformatic analysis

Sample quality control (QC) metrics of the raw reads were first calculated using DRAGEN FastQC plus MultiQC. The QC reports were generated for all samples. After passing QC, the raw reads were trimmed by Trimmomatic v.0.36 software to remove reads with technical bias, low-quality reads, and adapters. All raw reads were de novo assembled using SPAdes Genome Assembler v3.9.0 in auto K-mer mode. The assembled files were then subjected to gap closing using Rescaf software. The gap-filled assemblies were annotated via various bioinformatic tools.

### Genotyping E. Coli by multilocus sequence typing (MLST)

The sequence type of *E. coli* multilocus sequence typing scheme was performed using the nucleotide sequences of seven housekeeping genes, namely, *adk*, *fumC*, *gyrB*, *icd*, *mdh*, *purA*, and *recA*, according to the protocols available in the *E. coli* MLST database based on the method published by Larsen and others [30]. MLST was detected using the Achtman scheme with the MLST 2.0 (https://cge.food.dtu.dk/services/MLST/).

### Resistome analysis

The ResFinder-4.5.0 website [31] was also used to predict antimicrobial resistance genes in the next-generation sequencing data.

## Electronic supplementary material

Below is the link to the electronic supplementary material.

Supplementary Material 1

## Data Availability

The datasets used and/or analyzed during the current study are available from the corresponding author on reasonable request.
