# Supplementary material for: Isolation and evaluation of the pathogenicity of a hybrid shiga toxin-producing and Enterotoxigenic Escherichia coli in pigs
Source: BMC Vet Res. 2024 Oct 21;20:480. doi: 10.1186/s12917-024-04317-z (PMC11492512; doi:10.1186/s12917-024-04317-z)
Supplement: Supplementary file 1 — Supplementary Material 1 [file 12917_2024_4317_MOESM1_ESM.docx]

Supplementary Table 1. Primers used in multiplex and conventional PCR to detect *E. coli* virulence factors in this study

| Virulence factor | Nucleotide sequence (5’ to 3’) | Amplicon size | Reference |
| --- | --- | --- | --- |
| *STb*  STb-F  STb-R | TGCCTATGCATCTACACAAT CTCCAGCAGTACCATCTCTA | 113 | (20) |
| *STa*  STa-F  STa-R | CAACTGAATCACTTGACTCTT TTAATAACATCCAGCACAGG | 158 | (20) |
| *F5*  K99-F  K99-R | AATACTTGTTCAGGGAGAAA AACTTTGTGGTTAACTTCCT | 230 | (20) |
| *LT*   LT-F   LT-R | GGCGTTACTATCCTCTCTAT TGGTCTCGGTCAGATATGT | 272 | (20) |
| *F18*  F18-F  F18-R | TGGTAACGTATCAGCAACTA ACTTACAGTGCTATTCGACG | 313 | (20) |
| *F6*   987P-F   987P-R | GTAACTCCACCGTTTGTATC AAGTTACTGCCAGTCTATGC | 409 | (20) |
| *F4*   K88-F  K88-R | GAATCTGTCCGAGAATATCA GTTGGTACAGGTCTTAATGG | 499 | (20) |
| *F41*   F41-F  F41-R | AGTATCTGGTTCAGTGATGG CCACTATAAGAGGTTGAAGC | 612 | (20) |
| *STx2e*   STx2e-F  STx2e-R | AATAGTATACGGACAGCGAT TCTGACATTCTGGTTGACTC | 733 | (20) |
| Intimin (*Eae*)  Eae-F  Eae-R | GACCCGGCACAAGCATAAGC CCACCTGCAGCAACAAGAGG | 384 | (30) |

Supplementary Table 2. Protocol used in multiplex and conventional PCR for detection of *E. coli* virulence factors in this study

| Virulent genes | Initial denaturation | Denature | Annealing | Extension | Final extension | Cycles |
| --- | --- | --- | --- | --- | --- | --- |
| STb, STaP, F5, LT, F18, F6, F4, F41, and STx2e | 94 ^o^C  5 mins | 94 ^o^C  40 s | 55 ^o^C  1 min | 72 ^o^C  2 mins | 72 ^o^C  5 mins | 35 |
| Intimin (*Eae)* | 94 ^o^C  5 mins | 94 ^o^C  40 s | 55.9 ^o^C  1 min | 72 ^o^C  2 mins | 72 ^o^C  5 mins | 35 |

Supplementary Table 3. Antimicrobial susceptibility testing result of the hybrid Taiwan porcine STEC/ETEC isolate 2675 (No. 1)

| Antimicrobial agent | Interpretation | Antimicrobial agent | | Interpretation |
| --- | --- | --- | --- | --- |
| Amikacin | **S** | Enrofloxacin | R | |
| Amoxicillin | R | Tobramycin | R | |
| Ampicillin | R | Gentamycin | R | |
| Augmentin | R | **Imipenem** | **S** | |
| Azithromycin | R | Kanamycin | R | |
| Cefadroxil | R | Lincomycin | R | |
| Cefixime | R | **Meropenem** | **S** | |
| Cefovecin | R | Minocycline | R | |
| Ceftazidime | R | Neomycin | R | |
| Ceftiofur | R | Nitrofurantoin | I | |
| Cefuroxime | R | Norfloxacin | R | |
| Cephalexin | R | Ofloxacin | R | |
| Cephalothin | R | Penicillin G | R | |
| Cephazolin | R | Piperacillin | R | |
| Chloramphenicol | R | Polymyxin B | I | |
| Ciprofloxacin | R | Rifampicin | R | |
| Doxycycline | R | Tetracycline | R | |

S: susceptible; I:intermediate, R: resistant

Supplementary Table 4. Antimicrobial susceptibility testing result of the hybrid Taiwan porcine STEC/ETEC isolate 088 (No. 5)

| Antimicrobial agent | Interpretation |
| --- | --- |
| Enrofloxacin | R |
| Amoxicillin | R |
| Ampicillin | R |
| florfenicol | R |
| Gentamycin | R |
| Imipenem | **S** |
| flumquine | R |
| marbofloxacin | R |
| SXT | R |
| Ceftiofur | R |
| Cefquinome | **S** |
| Cefoperazone | **S** |
| Neomycin | R |
| Tetracycline | R |

S: susceptible; I:intermediate, R: resistant
